# Nijmegen Breakage Syndrome fibroblasts and iPSCs: cellular models for uncovering disease-associated signaling pathways and establishing a screening platform for anti-oxidants

**DOI:** 10.1038/s41598-017-07905-2

**Published:** 2017-08-08

**Authors:** Barbara Mlody, Wasco Wruck, Soraia Martins, Karl Sperling, James Adjaye

**Affiliations:** 1 Max-Delbrück-Centrum für Molekulare Medizin (MDC), 13092 Berlin, Germany; 2 Institute for Stem Cell Research and Regenerative Medicine, Medical Faculty, Heinrich Heine University, 40225 Düsseldorf, Germany; 3 Charité - Universitätsmedizin Berlin, Institute of Medical and Human Genetics, 13353 Berlin, Germany

## Abstract

Nijmegen Breakage Syndrome (NBS) is associated with cancer predisposition, premature aging, immune deficiency, microcephaly and is caused by mutations in the gene coding for NIBRIN (NBN) which is involved in DNA damage repair. Dermal-derived fibroblasts from NBS patients were reprogrammed into induced pluripotent stem cells (iPSCs) in order to bypass premature senescence. The influence of antioxidants on intracellular levels of ROS and DNA damage were screened and it was found that EDHB-an activator of the hypoxia pathway, decreased DNA damage in the presence of high oxidative stress. Furthermore, NBS fibroblasts but not NBS-iPSCs were found to be more susceptible to the induction of DNA damage than their healthy counterparts. Global transcriptome analysis comparing NBS to healthy fibroblasts and NBS-iPSCs to embryonic stem cells revealed regulation of P53 in NBS fibroblasts and NBS-iPSCs. Cell cycle related genes were down-regulated in NBS fibroblasts. Furthermore, oxidative phosphorylation was down-regulated and glycolysis up-regulated specifically in NBS-iPSCs compared to embryonic stem cells. Our study demonstrates the utility of NBS-iPSCs as a screening platform for anti-oxidants capable of suppressing DNA damage and a cellular model for studying NBN de-regulation in cancer and microcephaly.

## Introduction

Nijmegen Breakage Syndrome (NBS) is a rare autosomal recessive genetic disorder, first described 1981 in Nijmegen, the Netherlands^1^. Characteristics of NBS include genomic instability (resulting in early onset of malignancies), premature aging, microcephaly and other growth retardations, immune deficiency, impaired puberty and infertility in females. The consequence of these manifestations is a severe decrease in average life span, caused by cancer or infection of the respiratory and urinary tracts^2^.

On a molecular basis, NBS is caused by mutations in the gene coding for NIBRIN (*NBN*) which is involved in DNA damage repair^3^. Mutated versions of *NBN* cause accumulation of unrepaired DNA damage leading to cell cycle arrest, apoptosis^4^ or accumulation of genomic point mutations and aberrations introduced by misregulated DNA repair^5^. Several cases of NBS with a variety of mutations in *NBN* exist but over 90% of the patients carry a 5 base pair deletion (657del5) within the *NBN* exon 6^6^.

This hypomorphic mutation leads to a truncated 26 kD amino-terminal protein and a 70 kD carboxy-terminal protein due to alternative translation from a cryptic start site upstream of the deletion^7^. Mice *Nbn* null mutations are embryonic lethal and cells expressing only the truncated p26kD NBN fragment containing the FHA and the first BRCT domain, were nonviable^7^. The new splice form, p70 retains sufficient functionality to ensure survival by binding to MRE11 and ATM, which are essential components of DNA damage response^8^. The MRE11-RAD50-NBN (MRN) complex binds directly to DNA double-strand breaks (DSBs) and is involved in repair and signaling for homologous recombination (HR), non-homologous end joining (NHEJ) and microhomology-mediated end joining (MMEJ). Additionally, NBN is involved in telomere maintenance and therefore plays a role in the aging process^8^.

Recent works indicate that NBN influences the repair pathway choice via 53BP1, which can shift the error-free HR-directed repair to the more error-prone NHEJ and MMEJ^9^. Apart from replication errors, mutagens and other external influences, endogenously, DNA damage is mostly caused by reactive oxygen species (ROS), which are byproducts of the respiratory chain reaction^10^. Cells counteract ROS by antioxidant production and enzymatic removal but ROS also have cellular signaling functions which must be maintained in a controlled balance^11^.One strategy to minimize endogenous ROS levels is to regulate mitochondrial respiration, which plays a special role in stem cells.

Stem cell mitochondrial morphology is immature, rounded and with under-developed cristae. Consequently, they depend heavily on glycolysis for their ATP supply^12^. When cells differentiate and increase respiration, mitochondrial mass increases, their morphology then shifts to more matured and elongated tubular forms, with more defined cristae and increased mtDNA copy numbers^12^.

When somatic cells are reprogrammed into induced pluripotent stem cells (iPSCs), they depend predominantly on glycolysis and their mitochondria become rejuvenated and transformed back to the immature form^13^.

A key element in the reprogramming of metabolism is the HIF1-alpha pathway, which not only reacts in response to hypoxia, but also induces a shift from oxidative phosphorylation to glycolysis^14^. We have reported this “metabolic reprogramming” as an essential step in iPSC-generation, which precedes the activation of pluripotency-associated genes like OCT4 and NANOG^15^.

The aim of this study was to use our previously published iPSC-based cellular model system for NBS and provide a screening platform for antioxidants capable of modulating genome stability. NBS-iPSCs may overcome several problems associated with NBS research such as: i) small patient numbers, ii) cell cultures limited to fibroblasts and lymphocytes, iii) premature senescence in cell culture due to high levels of ROS, iv) discovery of new NBS molecular mechanisms and v) provision of new and therapeutically relevant concepts.

There are several diseases like NBS which derive from mutated genes in repair pathways, examples of these include Fanconi Anemia (FA)^16^, Ligase IV (LIG4) syndrome^17^,Bloom syndrome^18^,NBS-like disorder^19^, Ataxia-Telangiectasia-Like Disorder (ATLD)^20^, Nonhomologous end-joining factor 1 (NHEJ1) syndrome^21^ and Seckel syndrome^22^.

Our group recently published a study on modeling NBS by reprogramming^23^. Reprogrammed cells from patients with similar diseases like FA have been reported, though they could only be reprogrammed after genetic correction or with the aid of antioxidants^24^. In a study of patients with Cockayne syndrome (CS), a mutation in the repair pathway gene ERCC6 did not impair genetic reprogramming but exhibited elevated cell death rates and ROS production^25^. As NBS cells are hypersensitive to DNA damage^26^, ROS may be detrimental to them under physiological conditions. Thus, it was hypothesized that antioxidants or the induction of pluripotency in NBS fibroblasts might suppress and maybe bypass ROS-mediated genome instability.

Microcephaly is a significant physical characteristic of NBS which can also be found in FA, LIG4 and NHEJ1 syndromes^16, 20, 21^. With recent cases in microcephaly which coincided with infections of the Zika virus^27^, NBS-iPSCs and iPSC-derived neurons could serve as an excellent comparative model to study NBN-deregulation and associated molecular mechanisms underlying the onset of microcephaly.

In this study we present NBS fibroblasts and iPSCs as a screening platform for anti-oxidants and a model for studying NBN de-regulation in cancer and microcephaly. The screen for antioxidants capable of counteracting intracellular levels of ROS and DNA damage identified Ethyl-3,4-dihydroxybenzoate (EDHB) - an activator of the hypoxia pathway – as most potent antagonist of DNA damage in the presence of high oxidative stress in our NBS-model. Another finding was the higher susceptibility of NBS fibroblasts to induction of DNA damage compared to NBS-iPSCs. Furthermore, we found de-regulation of P53 in NBS fibroblasts and NBS-iPSCs, down-regulation of Cell cycle in NBS fibroblasts and down-regulation of oxidative phosphorylation and up-regulation of glycolysis in NBS-iPSCs compared to healthy embryonic stem cells.

## Results

### Roadblocks in reprogramming of NBS fibroblasts

Reprogramming of somatic cells towards pluripotent stem cells (PSCs) was reported to be negatively affected when P53 was activated^28^. Given the nature of NBS, which includes genomic instability and premature senescence, both of which are features known to lead to P53 activation^29^, thus, hurdles for the reprogramming process were anticipated. To address these, we attempted reprogramming dermal fibroblast primary cultures from eight (8) clinically diagnosed patients with NBS.

Of the 8 NBS fibroblast lines (Table 1), 3 lines could not be cultured past passage 6 and were lost due to premature senescence. Four (4) lines were infected with retroviral reprogramming cocktail (O/S/K/M) but exhibited low infection efficiency (determined by O/S/K/M immuno-staining), senescence (by morphology), hardly showed changes in morphology (negative indicator for reprogramming) and did not yield any iPSC colonies (data not shown).

**Table 1:** Fibroblasts lines from NBS patients and their behavior in reprogramming.

Cell line [NBS #]	Gender	Passage [#]	Premature senescence	NBN (657del5) Mutation
1	male	16	No	homozygous
2	male	5	Yes	homozygous
3	female	8	No	homozygous
4	female	14	Yes	homozygous
5	female	4	No	homozygous
6	female	10	Yes	homozygous
7	male	10	No	homozygous
8	male	3	No	heterozygous

n/a: not available; #: number.

As previously reported and characterized, only one of the four NBS fibroblast cell lines (NBS-8) subjected to the reprogramming process was successful^23, 30^. This shows that fibroblasts from NBS patients can be reprogrammed to pluripotency despite genomic instability and premature senescence^30^. Sanger-sequencing of *NBN* exon 6 and Western Blotting confirmed the heterozygous mutation for 657del5 in NBS-8 fibroblasts (Supplementary Figure S1) and in the NBS-8-iPS cells (Supplementary Figure S2). As the 657del5 mutation leads to a truncation of NBN on the protein level (wt: 95 kDa; 657del5: 70 kDa), we detected NBN by western blot. Full-length NBN was not present in any of the NBS fibroblasts including NBS-8 and the NBS-iPSCs^30^.

*In vitro* cultivation may introduce stress to the cells which may also elevate DNA double-strand breaks events. Accumulation of unrepaired damage DNA leads to the activation of P53, which is a known roadblock of induction of pluripotency as a result of cell cycle arrest, senescence or apoptosis^31^. Table 1 shows that the reprogrammable cell line NBS8 is heterozygous while the other cell lines are homozygous. It is probable that the homozygous nature of the mutated NBS gene increases the level of unrepaired damaged DNA hence drastically reducing the efficiency of inducing pluripotency. To examine this further, we performed transcriptome analysis of primary NBS fibroblasts cell cultures (1, 3, 5, 7 and 8).

Ingenuity® Pathway Analysis (IPA) was used to predict the status of transcription factors (TFs) using a list of differentially regulated genes between NBS and normal fibroblasts (Table 2). In the IPA results in Table 2 the fold change was determined by the expression data of the transcription factor itself but this can be different from the data inferred from the expression data of the de-regulated genes. The most significant, activated TF was P53 (TP53), well known to negatively interfere with reprogramming efficiency. We also found that the P53 pathway was significantly enriched in the transcriptomic analysis of the NBS fibroblasts (see “NBS-iPSCs as a model for studying molecular mechanisms associated with impaired DNA repair”). Among the most significant down-regulated or inactivated TFs was MYCN, which is known as transcriptional regulator in pluripotent stem cells^32^. This could also be a roadblock to reprogramming, as cells are required to proliferate continually during this process.

**Table 2:** Regulation changes in transcription factors in NBS fibroblasts (Ingenuity® Prediction Tool).

Transcription Regulator	Fold Change (Array data)	Predicted Activation State	Regulation z-score	Number of target molecules
TP53	1.48	Activated	4.32	175
CDKN2A	1.05	Activated	3.93	46
SMARCA4	−1.49	Activated	3.70	34
SMARCE1	1.24	Activated	2.45	6
TCF3	2.02	Activated	2.39	35
Rb (group)	n/a	Activated	2.26	14
GATA1	−1.09	Activated	2.24	11
TP63	−1.00	Activated	2.23	27
GLI3	1.65	Activated	2.11	4
SMAD7	−1.63	Inhibited	−2.13	16
SREBF1	−1.26	Inhibited	−2.31	22
RXRA	1.08	Inhibited	−2.46	17
SREBF2	1.16	Inhibited	−2.48	15
E2F1	1.36	Inhibited	−2.60	63
TBX2	−1.72	Inhibited	−2.77	24
MYCN	−1.07	Inhibited	−4.41	33

### Which genes/factors contribute to bypassing of cell cycle arrest, senescence or apoptosis

**Figure 1:**
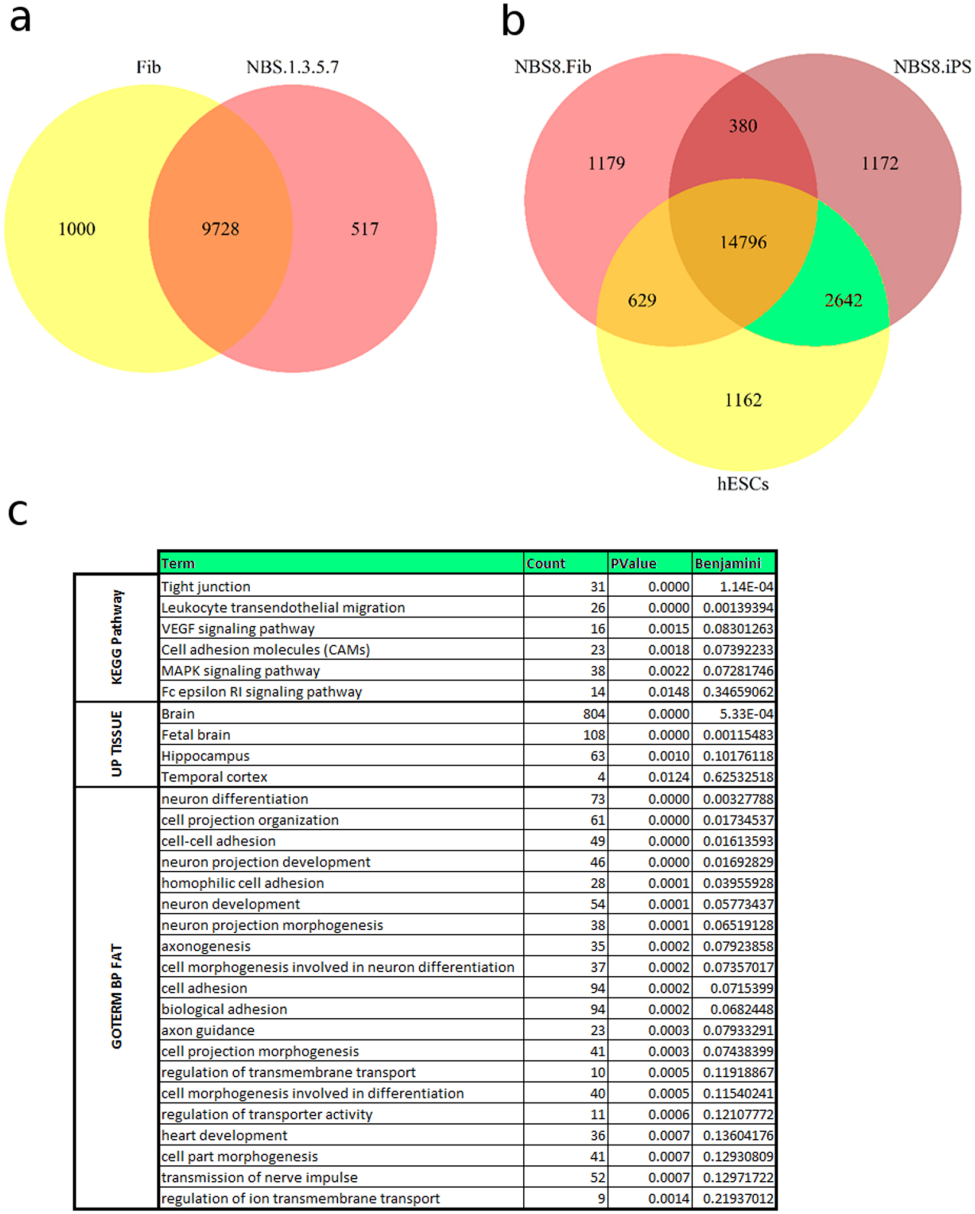
Statistics of Venn diagram analysis among NBS fibroblasts, NBS iPSCs and hESCs. The overlap of significantly expressed genes (detection p value < 0.01) in (**a**) Fibroblasts comparing the averaged group of NBS with unaffected cell lines and in (**b**) NBS fibroblasts versus NBS-iPSCs and unaffected hESCs. (**c**) Annotations for genes commonly expressed in NBS-iPSCs and hESCs but not NBS fibroblasts resulting from functional annotation analysis via the DAVID web tool. The output of the DAVID analysis was condensed to the *count* of genes annotated with the indicated category, *p-value* and *Benjamini-Hochberg*-correction for multiple-testing as calculated based on the Fisher-exact test.

To understand which mechanisms possibly enabled NBS fibroblasts to achieve pluripotency and therefore overcome cellular senescence, we compared transcriptomic data between NBS-8 fibs, NBS-8-iPSCs and hESCs. By identification of the overlaps for expressed genes (determined by expression p value < 0.01) we found 2642 genes to be commonly expressed in NBS-iPSCs and hESCs, but not in NBS fibroblasts (Fig. 1). This subset of genes may contain the distinct profile enabling NBS fibroblasts to reach pluripotency and therefore rejuvenation. We further analyzed the subset in an annotation database. Among the most over-represented results we found MAPK signaling pathway and genes that were specifically expressed in the brain (Fig. 1c). The MAPK signaling pathway is regulated by OCT4 and plays an important role in pluripotency and self-renewal^33^.

### Global transcriptome analysis of NBS-iPSCs

**Figure 2:**
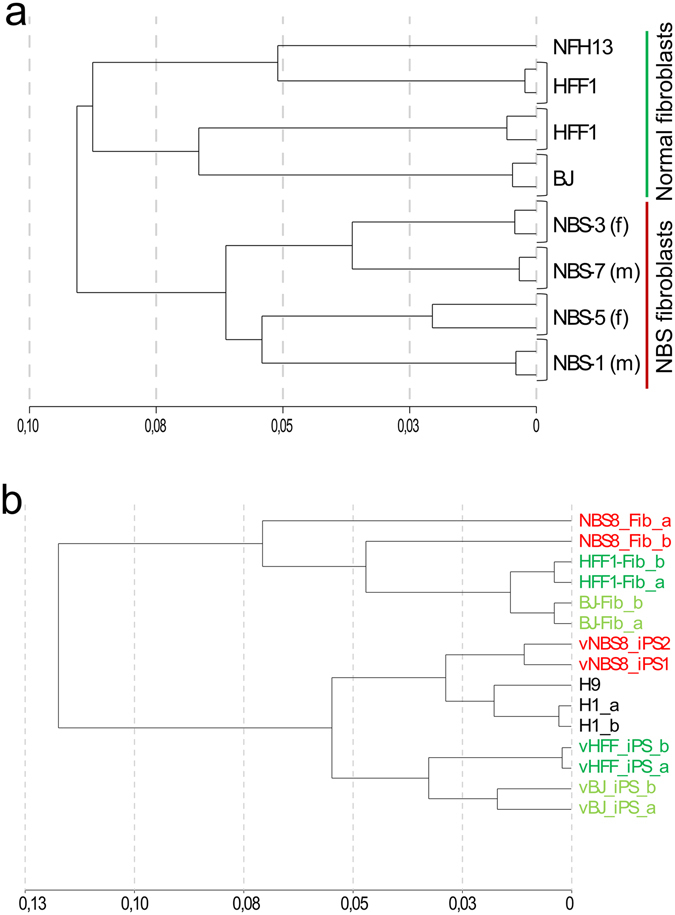
Global transcriptomic comparison of NBS fibroblasts and iPSCs to healthy controls. (**a**) Hierarchical cluster analysis depicting the distance of control (BJ, HFF1, NFH13) and patient (NBS-1, -3, -5, -7) fibroblasts global mRNA (Illumina 8-chip). (**b**) Hierarchical cluster analysis of total mRNA depicting the distance of control (BJ-Fib, HFF1-Fib) and patient (NBS8-Fib, passage number 8 and 15) fibroblasts, plus control (H1, H9, vHFF-iPS, vBJ-iPS) and patient (vNBS8-iPS, clone 1 and 2) pluripotent stem cells (Illumina 12-chip).

Global transcriptome analysis with NBS fibroblasts and NBS-iPSCs was performed to identify the problems interfering with reprogramming in the cell lines 1,3,5,7, determine NBS phenotypes or compensatory mechanisms in NBS-iPSCs derived from NBS-8 fibroblasts. In the cluster dendrogram (Fig. 2a), fibroblasts from NBS patients clearly clustered as a group and differed from normal fibroblasts, indicating a common transcriptional phenotype distinctive for NBS. Transcriptomes from NBS-iPSCs clustered closer to hESCs than to other fibroblasts-derived iPSCs (Fig. 2b). But, the parental fibroblast line NBS-8, clustered more distinct to the other fibroblasts. The pronounced gap in clustering between NBS-8-Fib-P8 and NBS-8-Fib-P15 indicates acquisition of mutations or aberrations since they only differ in passage number.

**Figure 3:**
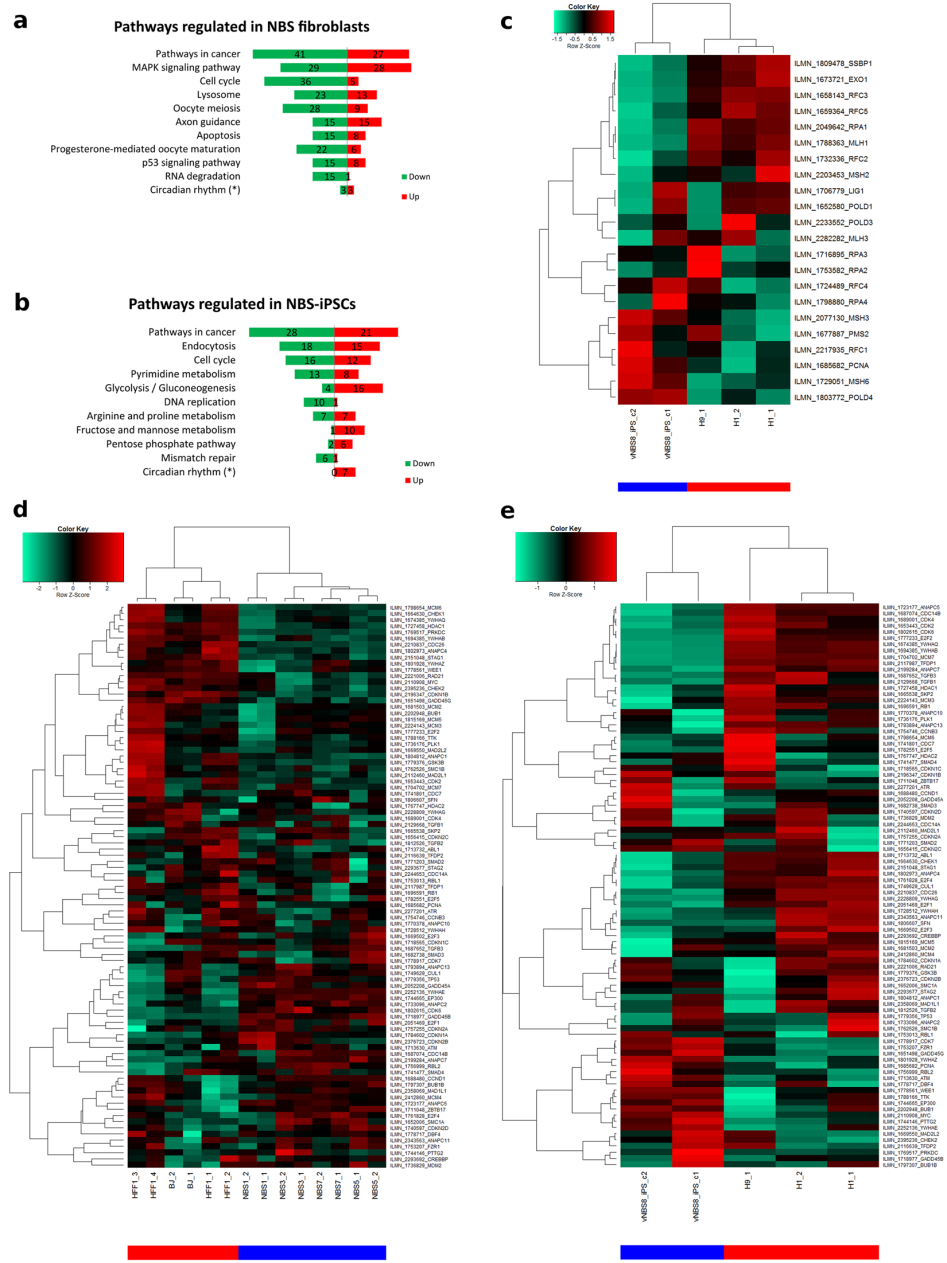
Functional transcriptomics study of NBS fibroblasts and iPSCs. Significantly de-regulated genes (differential p value < 0.05; fold-ratio > 1.5) between control and NBS-patient cells were analyzed by DAVID functional annotation tool (https://david.ncifcrf.gov/). The top 10 significantly (p value < 0.05) de-regulated KEGG pathways (http://www.genome.jp/kegg/pathway.html) are represented in the figure, numbers of significantly up-regulated genes are shown in red and down-regulated genes in green. (**a**) comparison between control (BJ, HFF1) and patient (NBS-1, -3, -5, -7) fibroblasts (**b**) comparison between control (H1, H9) and patient (vNBS8-iPS, clone 1 and 2) pluripotent stem cells. (*) p value of pathway > 0.05. The reprogramming procedure induced a shift of the Cell cycle pathway from down-regulated to nearly balanced. Furthermore, strong down-regulation of the Mismatch repair pathway was found in NBS-iPSCs. Cluster analysis and heatmaps of these de-regulated pathways are shown in (**c**–**e**): (**c**) depicts the Mismatch repair in NBS-iPSCs, (**d**) the Cell cycle in NBS fibroblasts and (**e**) the Cell cycle in NBS-iPSCs (Color bars: blue NBS, red control).

After selecting genes that were significantly de-regulated (p value < 0.05; fold change > 1.5) between the groups of NBS and normal fibroblasts, the list was subjected to “DAVID Annotation Tools”^34^ to identify pathways which were most affected by mutated NBN (Fig. 3a). The same procedure was performed for the analysis of NBS-iPSCs in comparison with hESCs (Fig. 3b). There were different regulatory changes in both groups of analyses (NBS fibroblasts and NBS-8-iPSCs), but there was also overlap of pathways, indicating NBS specific traits in cell cycle and cancer. *Apoptosis* and *P53*, two of the safeguard mechanisms against cancer, were predominantly de-regulated in NBS fibroblasts than pluripotent NBS cells while *Mismatch repair*, another safeguard mechanism against cancer, was predominantly de-regulated in pluripotent NBS cells. *Mismatch repair* is illustrated in more detail in the heatmap in Fig. 3c (color bars: blue = NBS, red = healthy). In *Cell cycle* we observed a shift from down-regulation to equally balanced (Fig. 3a,b,d,e).

Interestingly, in NBS-iPSCs, the *Glycolysis*-pathway was significantly enriched. Most enzymes involved in glycolysis, including phosphofructokinase, muscle (PFKM, 2-fold) which catalyzes the rate-limiting step, were at least 1.5-fold up-regulated (Supplementary Figure S3). On the other hand, Fructose-1,6-bisphosphatase 1 (FBP1), a gluconeogenesis regulatory enzyme, was significantly down-regulated (2.6-fold). As previously reported, hESCs derive their energy from glycolysis rather than OXPHOS and have immature mitochondria^13, 35^. It was also observed that cells acquire the same metabolic profile during the reprogramming process^13, 35^. NBS-iPSCs in this case, depended even stronger on glycolysis than other PSCs.

### NBS-iPSCs as a model for studying molecular mechanisms associated with impaired DNA repair

In line with the known predisposition of NBS patients to cancer, we found *Pathways in cancer* as the most enriched pathway in NBS-fibroblasts compared to healthy fibroblasts as well as in NBS-iPSCs compared to embryonic stem cells via DAVID analysis^34^ (Fig. 3a,b). With further cancer-related pathways, *Cell cycle* was found to be enriched in NBS-fibroblasts and NBS-iPSCs, while *Apoptosis* and *p53 signaling* were only enriched in NBS-fibroblasts. To further explore the relevance of the KEGG pathways in cancer^36^ we performed a further DAVID analysis with the genes annotated with that pathway in differentially expressed genes in NBS-iPSCs compared to embryonic stem cells and this way could refine the functional annotation of the *pathways in cancer* (suppl. Table. S2). As expected numerous specific cancer types such as lung cancer and Melanoma are annotated with these genes but also pathways related to NBS emerge. *Cell cycle* (Supplementary Figure S4) and *p53 signaling* (Supplementary Figure S5) are known to be impaired by NBS and are described in more detail in the section “Establishing the antioxidant screening platform”. The cluster analyses in Fig. 3 (c–e) and Supplementary Figure S6–S8 additionally provide a more detailed view of the dysregulation of dedicated genes in the pathways *Mismatch repair* (Fig. 3c), *Cell cycle* (Fig. 3d,e), *Glycolysis* (Supplementary Figure S6), *Oxidative phosphorylation* (Supplementary Figure S7) and *p53 signaling* (Supplementary Figure S8) between NBS and healthy states. The *Mismatch repair* (Fig. 3c) was predominantly down-regulated (6 of 7 genes) in the NBS-iPSCs. Impairment of *Mismatch repair* is associated with predisposition to cancer^37^. *Cell cycle* appears to shift from predominantly down-regulation (36 of 41 genes) in the NBS-fibroblasts to equal balance (16 genes down- and 12 genes up-regulated) in the NBS-iPSCs. Another shift along with the reprogramming took place from *Oxidative phosphorylation* in the NBS-fibroblasts to *Glycolysis* in the NBS-iPSCs (Supplementary Figures S6 and S7). This effect may have been induced by p53 down-regulation during reprogramming^38^ and showed similarities to the Warburg effect in cancer cells which produced energy by Glycolysis^39^. In line with our previous publication^23^, we found that essential genes such as TP53I3 in the p53 pathway shifted from up- to down-regulation during reprogramming. Supplementary Figure S8 depicts the *p53 signaling* in a comparison between NBS/WT PSCs and fibroblasts and demonstrates differences (including more down-regulation of the genes *P53* and *TP53I3)* in the only NBS line which could be reprogrammed (NBS8) compared to the other NBS lines.

### Establishing the antioxidant screening platform

**Figure 4:**
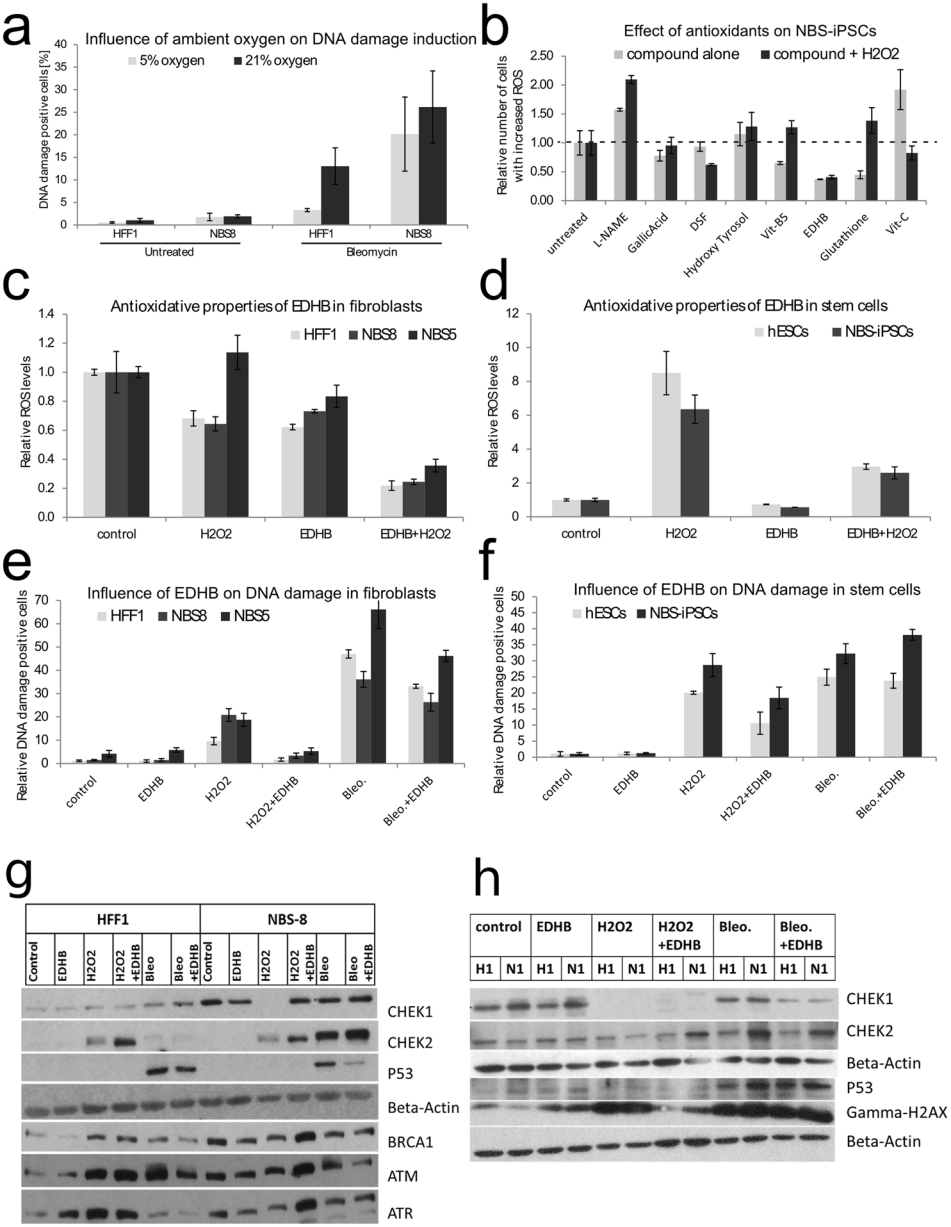
Response of NBS cells to oxidative stress and antioxidants. (**a**) The abundance of DNA damage measured by FACS-based detection of the DNA double-strand marker gamma-H2AX in HFF1 and NBS8 fibroblasts. DNA damage was induced by 30 µg/ml Bleomycin and compared under ambient (21%) and physiological (5%) oxygen concentrations. (**b**) NBS8-iPSCs were treated with either H_2_O_2_, several compounds known to influence DNA repair and ROS levels, or both. Internal ROS levels were then measured by FACS-based detection of the fluorescent ROS marker DCF-DA. The results were normalized to the untreated or peroxide-alone treated conditions respectively. (**c**) The influence of EDHB on internal ROS levels was tested on control (HFF1) and patient fibroblasts (NBS5, NBS8). The cells were either treated with EDHB alone or in combination with H_2_O_2_ to stimulate oxidative stress conditions. (**d**) Same experiment as in (**c**), but comparing control (hESCs) and patient (NBS-iPSCs) pluripotent stem cells. (**e**) The influence of EDHB on DNA damage (by detection of gamma-H2AX) was tested on control (HFF1) and patient fibroblasts (NBS5, NBS8). The cells were either treated with EDHB alone, in combination with H_2_O_2_ to stimulate oxidative stress conditions, or in combination with Bleomycin to stimulate mutagenic stress conditions. (**f**) Same experiment as in (**e**) but comparing control (hESCs) and patient (NBS-iPSCs) pluripotent stem cells. Bars indicate SD between independent experiments (*n* = 3). (**g**,**h**) Influence of DNA damage and EDHB on phosphorylation of DNA damage signaling proteins. Cells were treated with EDHB (antioxidant and inducer of HIFpathway), hydrogen peroxide (H_2_O_2_) and radiomimetic bleomycin (Bleo). (**g**) Immunofluorescent detection of phosphorylated signaling proteins in fibroblasts (HFF1, NBS-8) after SDS-PAGE. (**h**) Immunofluorescent detection of phosphorylated signaling proteins in hESCs (H1) and NBS-8-iPSCs (N1) after SDS-PAGE. Each lane of b-Actin corresponds to the lanes directly above and b-Actin is always unphosphorylated. For the sake of better readability western blots were cropped.

During cultivation of PSCs, which was performed at 5% oxygen levels, a temporary switch (12 h) to ambient (21%) oxygen resulted in apoptosis of NBS-iPSCs, but HFF1-iPSCs and hESCs were unaffected. We tested the effect of low (5%) and high (21%) oxygen quantitatively, by measuring gamma-H2AX, a marker for DNA double strand breaks in the presence or absence of the radiomimetic, Bleomycin. The result showed that low oxygen conditions greatly decreased the DNA damage under the influence of the mutagen. In addition, NBS Fibroblasts were more sensitive to DNA damage by mutagens than normal fibroblasts and the effect of low oxygen was less pronounced (Fig. 4a). NBS-iPSCs were then screened for various types of antioxidants to mimic or enhance the effect of low oxygen. The test measuring intracellular ROS levels revealed promising candidates to relieve NBS cells of oxidative stress, of which disulfiram (DSF) and EDHB were the most pronounced (Fig. 4b).

EDHB is utilized as a substrate analog and competitive inhibitor of prolyl 4-hydroxylases leading to specific inhibition of collagen synthesis^40^ and to activation of the hypoxia inducible factor (HIF)^41^. We tested the effect of EDHB on intracellular ROS levels under stress conditions by supplementation with hydrogen peroxide (H_2_O_2_). EDHB decreased normal ROS levels and greatly decreased intracellular ROS levels in the presence of H_2_O_2_ in fibroblasts and PSCs (Fig. 4c,d). Interestingly, in fibroblasts, ROS levels were higher in EDHB treatment alone compared to treatment with EDHB plus hydrogen peroxide while in PSCs EDHB alone was lower than that of EDHB plus hydrogen peroxide treatment group. This effect was even more pronounced in NBS cells and needs further exploration. One possible explanation would be a change in ROS levels due to the shift in energy supply from oxidative phosphorylation to glycolysis along with the reprogramming which was described in the above paragraph “Global transcriptome analysis of NBS-iPSCs”.

The effect of EDHB on DNA damage under stress conditions was also tested by supplementation with hydrogen peroxide or the radiomimetic Bleomycin. In fibroblasts, EDHB greatly decreased the DNA damage induced by hydrogen peroxide and moderately decreased the DNA damage caused by Bleomycin (Fig. 4e). In PSCs, EDHB decreased the DNA damage induced by H_2_O_2_ by 50%, but did not alter the DNA damage caused by Bleomycin (Fig. 4f). In addition, the same effects of EDHB, H_2_O_2_ and Bleomycin on DNA damage in PSCs could also be detected using western blotting (Fig. 4h).

As cells from patients with NBS are known to be affected by abnormal cell cycle checkpoints, e.g. failure of intra-S checkpoint after radiation^42^, we determined the influence of DNA damage (administered by Bleomycin-treatment) and HIF-Pathway activation by EDHB on the status of CHEK1 and CHEK2, which are usually phosphorylated upon activation^43^. CHEK1 (S345) phosphorylation is mostly facilitated by ATR and required for the G2/M DNA damage checkpoint^44, 45^. Upon DNA damage, CHEK1 becomes activated, it phosphorylates and inhibits CDC25C, thereby preventing activation of the cyclin B/CDK2 complex responsible for mitotic entry^46^. HFF1 cells only showed a slight increase in P-CHEK1 after treatment with bleomycin, but NBS-8 fibroblasts on the other hand exhibited CHEK1 activation without any treatment (Fig. 4g). This was decreased, but not eliminated upon treatment with EDHB. Bleomycin did not cause stronger activation than in control and this was not challenged by EDHB treatment. Interestingly, the supplementation with H_2_O_2_ completely diminished CHEK1 phosphorylation but not in the presence of EDHB.NBS iPSCs were found to have slightly higher base-level of phosphorylated CHEK1 in comparison to unaffected PSCs (H1, Fig. 4h). Treatment with EDHB reduced P-CHEK1 levels in NBS iPSCs under radiomimetic stress (Bleomycin) conditions, compared to the control.

CHEK2 is known to be phosphorylated (T68) and activated in an ATM-dependent manner in response to ionizing radiation^47^. In our NBS-context it is essential that the MRN complex regulates the activation of ATM^43, 48^ and acts upstream as well as downstream of ATM^49^. Activated CHEK2 phosphorylates P53 at serine-20^50^ CDC25A at serine-123^51^ and CDC25C at serine-216 thus, contributing to the G1/S, S, and G2/M checkpoints respectively^52^. In HFF1 cells treated with H_2_O_2_, CHEK2 became phosphorylated and this increased after combined application of H_2_O_2_ and EDHB (Fig. 4g). The same effect was observed in NBS-8 fibroblasts. Bleomycin did not activate CHEK2 in HFF1 cells, but the activation was strong in NBS-8 cells. Here, a low base-level of P-CHEK2 in PSCs was observed (Fig. 4h). Upon DNA damage, P-CHEK2 activation was significantly high in NBS-iPSCs (N1) but decreased after treatment with EDHB (Fig. 4h).

Previous experiments using FACS analysis revealed that EDHB can decrease DNA damage caused by H_2_O_2_ in fibroblasts and iPSCs, but only moderately reduce the damage in fibroblasts caused by bleomycin (data not shown). Western Blot analysis of gamma-H2AX after treating hESCs and NBS-8-iPSCs with H_2_O_2_ and bleomycin confirmed these measurements (Fig. 4h). It also showed that the application of H_2_O_2_ can indeed result in DSBs as indicated by the increased detection of gamma-H2AX. But it is important to keep in mind that in comparison (y-H2AX measurement, (Fig. 4h), H_2_O_2_ induced approx. the same level of DNA damage in hESCs as bleomycin, but 2-fold lower DNA damage in fibroblasts than in ESCs. Here, DNA damage was induced by oxidative stress in the form of H_2_O_2_ and by the DSB-inducer bleomycin (Fig. 4g,h).

This study shows, that in HFF1 cells, P53 S15 phosphorylation was observed after treatment with bleomycin, but not after H_2_O_2_ administration. In addition, the level of induction was lowered by addition of EDHB in the bleomycin treatment. In NBS-8 cells, the same effect was observed, but the P53 activation by bleomycin was lower and was almost abolished after EDHB treatment (Fig. 4g). In hESCs and NBS-8-iPSCs P53 was only phosphorylated at S15 after bleomycin treatment as well. But here, P-P53 was higher in NBS-8-iPSCs and EDHB treatment did not show a clear effect (Fig. 4h).

ATR is activated by ssDNA that result at a later stage in homologous recombination repair (HRR), or result from stalled replication forks. In NBS, repair from HR is impaired, so ATR signaling is rather activated by stalled replication forks. In NBS-8 cells, phosphorylated ATM exhibited a similar level without or after treatment with H_2_O_2_ or bleomycin (Fig. 4g). EDHB on the other hand increased the signal in cells treated with H_2_O_2_ (oxidative stress) and decreased the signal in cells treated with bleomycin (DSB inducer). Control cells, HFF1, exhibited lower basal levels of P-ATM than in NBS-8 cells but got strongly activated after H_2_O_2_ and bleomycin treatment. EDHB decreased the ATM activation by bleomycin as well. In contrast to ATM, ATR was not activated in HFF1 and NBS-8 cells by bleomycin. It was phosphorylated after treatment with EDHB or H_2_O_2_ in HFF1 cells, but not in NBS-8 cells. Here it only became activated after treatment with H_2_O_2_ and EDHB together. Comparison of ATM activation with activation of its target CHEK2, did not show the expected similar expression level in the western blot, neither did CHEK1 as target of ATM.

Here, BRCA1 S1524 phosphorylation appeared on a similar level of activation as ATR in NBS-8 cells, but different in HFF1 and also different in both cases in comparison to ATM (Fig. 4g). In detail, BRCA1 was slightly activated by H_2_O_2_ and bleomycin in HFF1 cells with no difference after addition of EDHB in any case. Again, there was a high level of phosphorylated protein in the control in NBS-8 cells, which was only further raised after treatment with H_2_O_2_ and EDHB. EDHB alone, H_2_O_2_ alone, bleomycin alone and bleomycin plus EDHB did show a similar expression of P-BRCA1, which was lower than in control.

The important finding here is the ability of EDHB to decrease the amount of DSBs caused by oxidative stress (administered by H_2_O_2_). EDHB showed a reduction of P-CHEK1 and P-CHEK2 in hESCs and iPSCs after bleomycin treatment, and an induction of P-CHEK1 and P-CHEK2 in fibroblasts after treatment of H_2_O_2_. Interestingly, the important tumor suppressor TP53 was less activated in NBS-8 fibroblasts as in control cells (HFF1) and exhibited an even lower signal after treatment with bleomycin.

The pattern in activation of P53, CHEK1 and CHEK2 is similar, but not identical in NBS-8 fibroblasts and iPSCs and differs in comparison to their healthy counterparts. The most prominent difference is the response of CHEK1 to EDHB in NBS-8 fibroblasts in comparison to NBS-8-iPSCs (activated in response to H_2_O_2_ only in the presence of EDHB). Furthermore, the relative activation of CHEK2 to bleomycin is a lot stronger in NBS-8 fibroblasts compared to iPSCs.

## Discussion

The DNA damage sensing *NBN* is an adapter protein which can bind to a variety of other DNA signaling and repair proteins particularly ATM, which is a kinase that amplifies and transduces the DNA damage signal^53^. The 657del5 mutation in NBN results in a truncated protein where one specific functional domain (FHA-BRCT) is missing. This domain is also a common motif within other DNA repair signaling proteins^54^. DNA repair mechanisms especially those of repair pathway decisions are still not fully understood. NBS-iPSCs and their differentiated descendants could therefore serve as a good model to study DNA repair and cell fate after DNA damage. This could aid in elucidating the mechanisms underlying the disease. NBS-iPSCs can also provide a screening system for treatments which might increase the life span and quality of life of patients with NBS and similar diseases like Fanconi Anemia (FA), Ligase IV (LIG4) syndrome, Bloom syndrome, NBS-like disorder, ataxia-telangiectasia-like disorder (ATLD), Nonhomologous end-joining factor 1 (NHEJ1) syndrome and Seckel syndrome, which all derive from mutated genes in repair pathways^55, 56^. In a recent publication, we modeled and characterized NBS by reprogramming^23^. Reprogrammed cells from patients with similar diseases like FA have been reported, though this can be done only after genetic correction or with the aid of antioxidants^24^. In another study, a mutation in the repair pathway gene ERCC6 did not prevent genetic reprogramming but exhibited elevated cell death rates and ROS production^25^.

Our cellular NBS model was based on fibroblasts from NBS patients reprogrammed into iPSCs, using retroviral transduction of OCT4, SOX2, KLF4 and C-MYC. Further, by employing somatic cells and iPSCs of NBS, global transcriptome analysis was performed, to identify new phenotypes and changes in the signaling network of NBS cells compared to normal cells. In addition, the influence of oxidative stress, radiomimetics and antioxidants was tested on the genomic integrity of NBS cells before and after reprogramming. Comparative transcriptome and associated pathway analyses revealed that, (a) NBS fibroblasts have a higher impact on cell cycle regulation, apoptosis and P53 signaling than normal fibroblasts (b) NBS-iPSCs and normal hESCs presented de-regulated genes and pathways associated with DNA replication, glycolysis, pyrimidine, fructose and mannose metabolism as well as DNA repair related pathways. Notably, these pathways can be connected to ROS homeostasis. Comparative tests based on sensitivity towards oxidative stress and DNA damaging agents such as hydrogen peroxide and Bleomycin, revealed that NBS-iPSCs and NBS-fibroblasts compared to normal fibroblasts were highly sensitive to DSB inducer Bleomycin and oxidative stress induced by exogenous hydrogen peroxide. Interestingly, DNA damage from hydrogen peroxide was efficiently relieved by addition of EDHB, an inducer of the hypoxia (HIF) pathway. The results indicate that NBS-iPSCs can serve as an excellent model to study NBS and screen for antioxidants *in vitro*.

NBS is a disease of premature aging resulting from the genomic instability caused by the *NBN* mutation which leads to hurdles in the reprogramming process. Activation of P53 is especially known to restrain reprogramming^28^. We observed that P53, a known tumor-suppressor gene, was activated in NBS fibroblasts, resulting in increased senescence in the NBS cell cultures and had extremely low reprogramming efficiency. Once reprogrammed, the cells maintained pluripotency and proliferated like normal hPSCs. NBS-iPSCs may protect themselves from oxidative stress and ROS-induced DNA damage by increased glycolysis which was up-regulated in comparison to hESCs. In previous studies, hESCs were found to have immature mitochondria and depended heavily on glycolysis^13, 15, 35, 57^. This bias towards glycolysis might be related to the down regulation of P53, increased stress in the NBS iPSCs and hence increased glycolytic lactate production for survival. Furthermore, it is known that P53 promotes oxidative phosphorylation^38^, thus, reduced P53 results in reduced oxidative phosphorylation.

Also, PSCs are known to ensure genomic integrity through enhanced apoptosis induction and increased antioxidant defense, contributing to protection against DNA damage^58^. The finding that antioxidants, particularly EDHB, improved genomic stability of NBS-iPSCs can improve reprogramming of additional NBS fibroblasts and other diseases like NBS which derive from mutated genes in DNA repair pathways, examples include, Fanconi Anemia (FA)^16^, Ligase IV (LIG4) syndrome^17^, Bloom syndrome^18^, NBS-like disorder^19^, Ataxia-Telangiectasia-Like Disorder (ATLD)^20^, Nonhomologous end-joining factor 1 (NHEJ1) syndrome^21^ and Seckel Syndrome^22^.

EDHB was used in another study to protect cells from hypoxia-mediated oxidative damage^59^. With EDHB known as an activator of the HIF pathway, these results point to a reduction of ROS-induced DNA damage and subsequent relief of the impaired DNA damage response as the cause for genomic stabilization.

DNA damage response mediated by the MRN complex, ATM/ATR, P53, CHEK1 and CHEK2 are crucial in early development of most types of cancer^60^. Although the role of this core network in relation to DNA damage, cancer and pluripotency has been widely investigated^31, 43^, several mechanisms in early oncogenesis remain unclear.

In this study, we have demonstrated that our model of fibroblasts and iPSCs derived from NBS patients besides the study of NBS itself which is associated with microcephaly, premature aging and growth retardation provides the environment for a detailed study of oncogenic mechanisms. The NBS phenotype includes a predisposition to cancer due to impaired DNA damage repair. Furthermore, we have shown that the addition of stimuli such as oxidative stress and mutagenic factors to this model could be used as a screening platform for anti-oxidants capable of suppressing DNA damage. Transcriptome analysis of our NBS-model identified de-regulation of P53, cell cycle, oxidative phosphorylation and glycolysis. In screening for antioxidants we identified EDHB as a potent modulator of DNA damage. Interestingly we revealed that NBS fibroblasts have a higher susceptibility for induction of DNA damage compared to NBS-iPSCs. However, although additional research is needed to improve the reprogramming efficiency and thus the robustness we believe that NBS-iPSCs can serve as cellular tools for a screening platform for molecules with anti-oxidant capabilities.

## Methods

### Ethical approval

NBS patient dermal fibroblast cells with informed consent (Table 1) were provided by Prof. Dr. Karl Sperling (Institute of Medical and Human Genetics, Charité - Universitätsmedizin Berlin, 13353 Berlin). Approval was obtained from the Ethics Commission of the Charité—Universitätsmedizin. The methods and experimental protocols were carried out in accordance with their guidelines and regulations.

### Cell culture

Neonatal foreskin fibroblasts, HFF1 and BJ were purchased from ATCC (#SCRC-1041 and #CRL-2522, respectively). All cells used were cultured at 37 °C, 5% CO_2_ and either 21% (standard) or 5% oxygen in an incubator under humidified atmosphere. Somatic cells were cultured in DMEM medium (Gibco, USA) supplemented with 10% fetal bovine serum (FBS) and 1x penicillin/streptomycin until reaching 90% confluency and then split in a 1:4 ratio. The conditions for passaging human pluripotent stem cells (hPSC) were a combination of methods adapted from several published protocols^61, 62^. This was applied to the culture of the human ESC-lines H1 and H9 (WiCell Research Institute, Madison, WI, USA) and iPSCs generated from NBS and HFF1 cells. In combination with MEFs, hPSCs were usually cultivated in plates coated with 0,2% gelatin and fed with hESC medium containing KO-DMEM supplemented with 20% knockout serum replacement, non-essential amino acids, L-glutamine, penicillin/streptomycin, sodium pyruvate, 0.1 mM beta-mercaptoethanol and 4 ng/ml FGF-2, which was replaced every second day.

### FACS analysis (detection of ROS and DNA damage)

The FACSCalibur system (BD Biosciences, USA) and the software program CellQuestPro were used as described by the manufacturer’s instructions. Programs used for data analysis were CellQuestPro (BD Biosciences, USA), Cyflogic (Cyflo Ltd, Finland), Weasel 3.0 (WEHI, Australia) and Flowing software 2.5.1 (Finland) programs.

For ROS measurement, fibroblast cells were seeded onto 12-well-plates with a density of 5 × 10^4^ cells per well one day prior to treatment. hESCs and iPSCs were seeded on Matrigel (Corning) in 6-well-plates and fed with hESC medium, one week prior to the treatment. To prepare cells for ROS-measurements, they were washed once with PBS and then incubated in 15 µM DCF-DA for 20 min at 37 °C. Afterwards, the solution was removed and the cells were briefly rinsed with PBS. Cells were treated with different concentrations of antioxidants (as indicated) and/or 20 µM H_2_O_2_ for 30 min. To analyze single cells by FACS, they were trypsinized by colorless 0.05% trypsin solution for 5 min. Trypsinization was stopped by adding 10% FBS in PBS. Cells were then centrifuged by 500 × g for 5 min and re-suspended in 300 µl PBS. The fluorescence was measured by FACS using the FITC channel.

For measurement of DNA damage, fibroblast cells were seeded onto 6-well-plates with a density of 4 × 10^5^ cells per well one day prior to treatment. hESCs and iPSCs were seeded on Matrigel (Corning) in 6-well-plates and fed with hESC medium, one week prior to the treatment. Cells were either treated with antioxidants (as indicated) 5 min prior to the addition of 1 mM H_2_O_2_ or with H_2_O_2_ alone at a total incubation time of 4 h at 37 °C in a cell culture incubator with either 21% or 5% oxygen, as indicated. Other cells were treated with antioxidants (as indicated) 5 min prior to the addition of 30 µg/ml Bleomycin for 3 h, and released for 1 h by switching to Bleomycin-free medium. Afterwards, the cells were briefly rinsed with PBS and trypsinized to generate single cells. The cells were centrifuged at 500 × g for 5 min and the cell pellet was re-suspended in 100 µl PBS. Under constant shaking (to prevent clumping) 300 µl of 100% ice-cold ethanol was added dropwise to fix the cells and incubated at −20 °C for at least 30 min or until further use. Afterwards the solution was mixed with 1 ml PBS and centrifuged at 2200 × g for 5 min. The pellet was re-suspended in 50 ml PBS-T with 5% FBS and incubated 30 min at RT for blocking. FITC-labeled gamma-H2AX antibody (Millipore, 1:500) was added and incubated overnight at 4 °C. The next day, 300 µl PBS was added and the cells were measured by FACS using the FITC channel. In some cases, the cells were co-stained with TRA1-60 antibody (Santa Cruz Biotechnology, Inc.) to verify pluripotent cell populations.

### Western Blot

The membrane was rinsed with dH_2_O and then blocked with 5% milk powder or 5% BSA in PBS-T (blocking solution) by shaking for 1 h at RT. BSA blocking solution was used for phospho-specific antibodies, in all other cases blocking was performed with milk. After blocking, the membrane was incubated by shaking at 4 °C overnight with the primary antibodies dissolved in PBS-T with 5% milk powder or 5% BSA; Beta-actin (Sigma-Aldrich), phospho-histone H2A.X (Ser139), phospho-CHEK1 and phospho-CHEK2 (CST), phospho-P53 (Ser15, CST), phospho-BRCA1 (Ser1524, CST), phospho-ATM (Ser1981, CST), phospho-ATR (Ser428, CST). Afterwards, the membrane was washed 3 times for 10 min in PBS-T on the Lab shaker, exchanging buffer between each step. Then, the secondary antibody dissolved in milk or BSA blocking solution was applied by shaking for 1 h at RT. Afterwards the membrane was washed 3 times for 10 min in PBS-T. Appropriate peroxidase-conjugated secondary antibodies and luminescence was induced by ECL Plus Western Blotting Detection Reagents and captured on BioMAX XAR film.

### Transcriptomics

The microarray hybridization experiments included biotin-labelling of cRNA by using 500 ng quality-checked total RNA (per sample) as input. Chip hybridizations, washing, Cy3 streptavidin staining, and scanning were performed on BeadStation 500 platform (Illumina) using reagents and protocols supplied by the manufacturer. cRNA samples were hybridized in duplicates on Illumina human-8 BeadChips (NBS-1, NBS-3, NBS-5, NBS-7, HFF1, BJ) or Illumina human-12 BeadChips (NBS-8, NBS-8 iPSCs, H1, H9 (single), HFF1-iPSCs, BJ-iPSCs). Basic expression data analysis was carried out using the manufacturer’s software GenomeStudio (Illumina). Raw data was background-subtracted and normalized using the “rank invariant” algorithm. Normalized data was then filtered for significant expression (detection p-value) based on negative control beads. All genes with detection p-values below 0.01 were considered as expressed. All genes with differential p-values below 0.05 were considered as differentially expressed. Selection for differentially expressed genes was performed on the basis of arbitrary thresholds (1.5 fold changes) and statistical significance according to an Illumina custom model^63^. Different sets of gene lists were entered into the DAVID functional annotation tool^34^, using the official gene symbol or ILLUMINA-IDs as input, to perform gene-annotation enrichment analysis, functional annotation clustering, KEGG pathway mapping (http://www.genome.jp/kegg/)^36^, transcription factor binding site prediction and more. Multiple testing was assessed via the Benjamini-Hochberg correction in the results of the DAVID analysis.

For the calculation of the activation state of transcription factors, a list of differentially regulated genes between NBS and normal fibroblasts was used as input for Ingenuity® Pathway Analysis (IPA®, QIAGEN Redwood City, www.qiagen.com/ingenuity).

Microarray data is available at NCBI GEO under the accession number GSE94708 for the superseries and GSE94706 for the fibroblasts and GSE94707 for the iPSCs series.

## Electronic supplementary material

Supplementary Information
