# Supplementary material for: Nijmegen Breakage Syndrome fibroblasts and iPSCs: cellular models for uncovering disease-associated signaling pathways and establishing a screening platform for anti-oxidants
Source: Sci Rep. 2017 Aug 8;7:7516. doi: 10.1038/s41598-017-07905-2 (PMC5548734; doi:10.1038/s41598-017-07905-2)
Supplement: Supplementary file 1 — Supplementary Information [file 41598_2017_7905_MOESM1_ESM.pdf]

Supplementary Information to “Nijmegen Breakage Syndrome fibroblasts and iPSCs: cellular models for uncovering disease-associated signaling pathways and establishing a screening platform for anti-oxidants” by Barbara Mlody, Wasco Wruck, Soraia Martins, Karl Sperling and James Adjaye

Supplementary figures legends

Supplementary Figure S1: Characterization of the NBS mutation. (Modified from our previous publication [1]) a) PCR-based analysis of genomic DNA with primers flanking the deletion, results in either a 60 bp (wt) or 55 bp (657del5) amplicon. b) Sanger sequencing results comparing the genomic DNA sequence of fibroblast sample NBS8 against HFF1 fibroblasts (normal), on the top the blasting result (http://blast.ncbi.nlm.nih.gov/Blast.cgi) from HFF1 Exon 6 (fw) against NBN mRNA (NM_002485.4), then NBS 1 Exon 6 (fw) against HFF1 Exon 6 (fw) and NBS 8 Exon 6 (fw) against HFF1 Exon 6 (fw). Below the chromatogram of the sequence is shown. c) Immunoflurescent detection of full-length (p95) NBN in patient fibroblasts. Even loading of the SDS-gel is shown by house-keeping protein GAPDH. d) Western Blot detection of p95-NBN in reprogrammed cells. ß-Actin serves as a loading control. For the sake of better readability western blots were cropped.

Supplementary Figure S2: Sanger sequencing confirms the NBS causing deletion 657del5 in the NBS-iPSCs. Results of Sanger sequencing of the NBS8-iPSCs showing the chromatogram of the region around the heterozygous 657del5 deletion in the *NBS* gene.

Supplementary Figure S3: NBS-iPSCs: Differentially regulated genes involved in *Glycolysis*. KEGG pathway (hsa00010)[2] of *Glycolysis/Gluconeogenesis*. In red: up-regulated; in green: down-regulated genes in NBS-iPSCs vs. control, classified by differential p-value < 0.05 and fold-change > 1.5.

Supplementary Figure S4: NBS-iPSCs vs. ESCs: Differentially expressed genes from *Pathways in cancer and Cell cycle.* Genes differentially expressed in NBS-iPSCs vs. control, classified by differential p-value < 0.05 and fold-change > 1.5 and functionally annotated as *Pathways in cancer* were further refined by another DAVID analysis. Resulting genes annotated with the KEGG pathway *Cell cycle*[2] were mapped to the chart of this pathway.

Supplementary Figure S5: NBS-iPSCs vs. ESCs: Differentially expressed genes from *Pathways in cancer and p53-signaling.* Genes differentially expressed in NBS-iPSCs vs. control, classified by differential p-value < 0.05 and fold-change > 1.5 and functionally annotated as *Pathways in cancer* were further refined by another DAVID analysis. Resulting genes annotated with the KEGG pathway *p53-signaling*[2] were mapped to the chart of this pathway.

Supplementary Figure S6: Reprogramming of NBS fibroblasts into iPSCs shifts energy supply to *Glycolysis*. Hierarchical cluster analysis and heatmap of regulated genes from the KEGG pathway *Glycolysis*[2] in a) NBS fibroblasts and b) NBS-iPSCs shows a change in the *Glycolysis* pathway from predominantly down-regulated in NBS fibroblasts to predominantly up-regulated in NBS-iPSCs. The

sample clustering displays a clear separation of NBS and healthy control clusters. (Color bars: blue NBS, red control; high expression: red, low expression; green).

Supplementary Figure S7: Cluster analysis of *Oxidative phosphorylation* pathway in NBS fibroblasts and iPSCs. Hierarchical cluster analysis and heatmap of genes from the KEGG pathway *Oxidative phosphorylation*[2] in a) NBS fibroblasts and b) NBS-iPSCs shows clear separation of NBS and healthy control clusters. If at all, there is only a slight tendency of more down-regulation of the NBS resulting from the reprogramming because the predominantly down-regulated cluster is bigger in the NBS-iPSCs than in the NBS fibroblasts. (Color bars: blue NBS, red control; high expression: red, low expression; green).

Supplementary Figure S8: Cluster analysis of *p53 signaling* pathway in NBS fibroblasts and iPSCs. Hierarchical cluster analysis and heatmap of genes from the KEGG pathway *p53-signaling*[2] in NBS fibroblasts and NBS-iPSCs show a cluster of pluripotent stem cells and another cluster of fibroblasts. Within these clusters NBS and healthy control samples were separated. Passage 8 and 15 of the only NBS line which could be reprogrammed showed differences to the other NBS fibroblast lines (Color bars: blue NBS, red control; high expression: red, low expression; green).

References

1. Mlody, B. & Adjaye, J. Generation of iPSC lines from a Nijmegen Breakage Syndrome patient. *Stem Cell Res.* **15,** 629–632 (2015).

2. Kanehisa, M., Furumichi, M., Tanabe, M., Sato, Y. & Morishima, K. KEGG: new perspectives on genomes, pathways, diseases and drugs. *Nucleic Acids Res.* **45,** D353–D361 (2017).

Supplementary figures

Supplementary Figure S1: Characterization of the NBS mutation

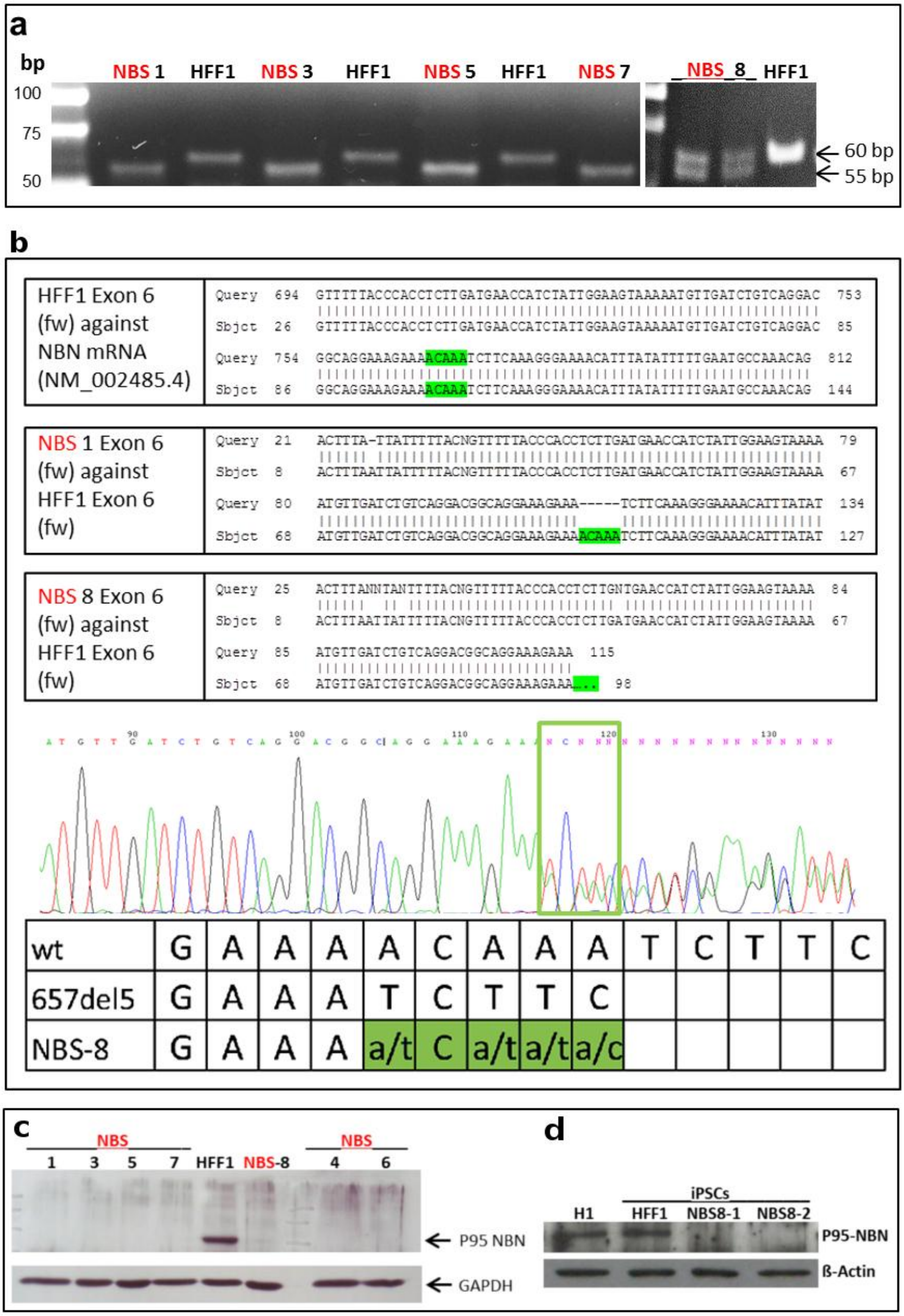

Supplementary Figure S2: Sanger sequencing confirms the NBS causing deletion 657del5 in the NBS-iPSCs

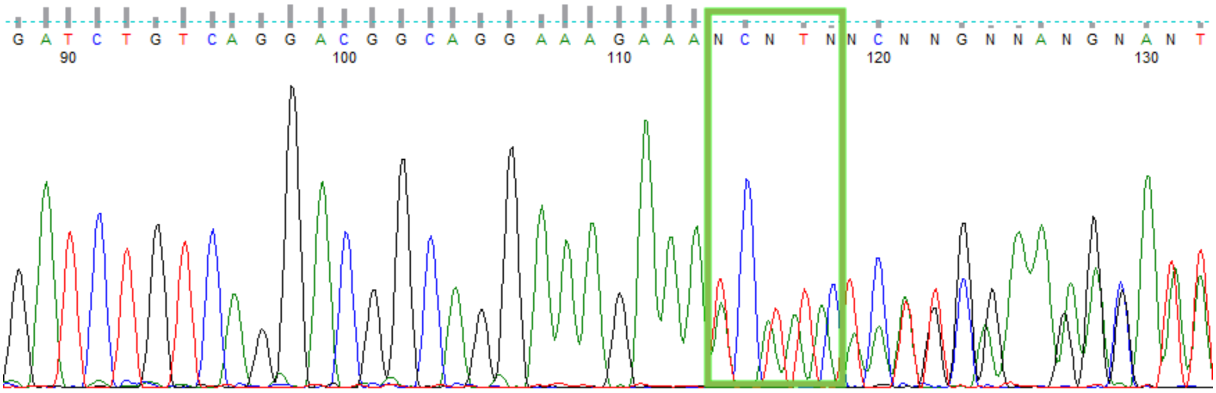

wt	G	A	A	A	A	C	A	A	A	T	C	T	T	C
657del5	G	A	A	A	T	C	T	T	C					
NBS8-iPSC	G	A	A	A	a/t	C	a/t	a/t	a/c					

Supplementary Figure S3: NBS-iPSCs: Differentially regulated genes involved in *Glycolysis*

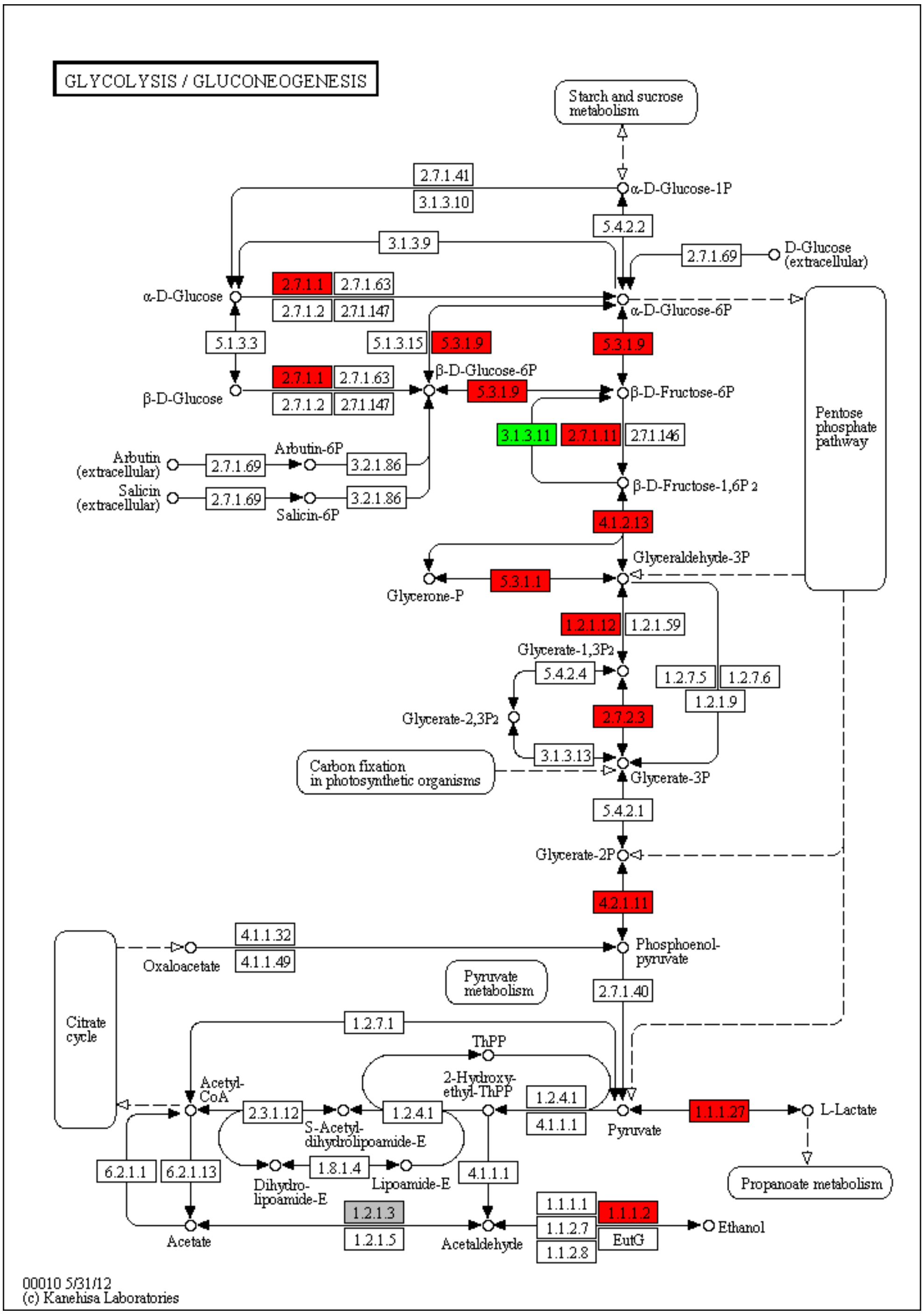

Supplementary Figure S4: NBS-iPSCs vs. ESCs: Differentially expressedgenes from *Pathways in cancer and Cell cycle*

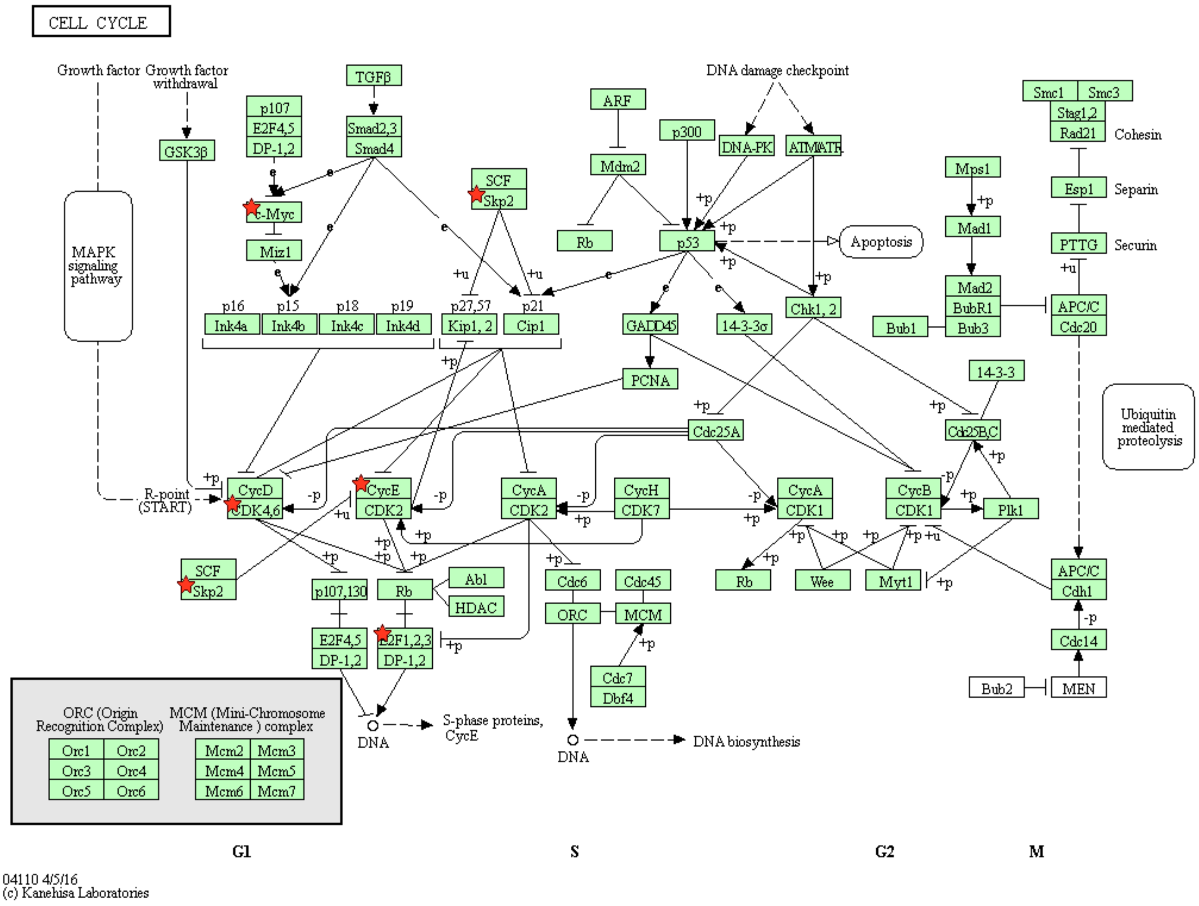

Supplementary Figure S5: NBS-iPSCs vs. ESCs: Differentially expressed genes from *Pathways in cancer and p53-signaling*

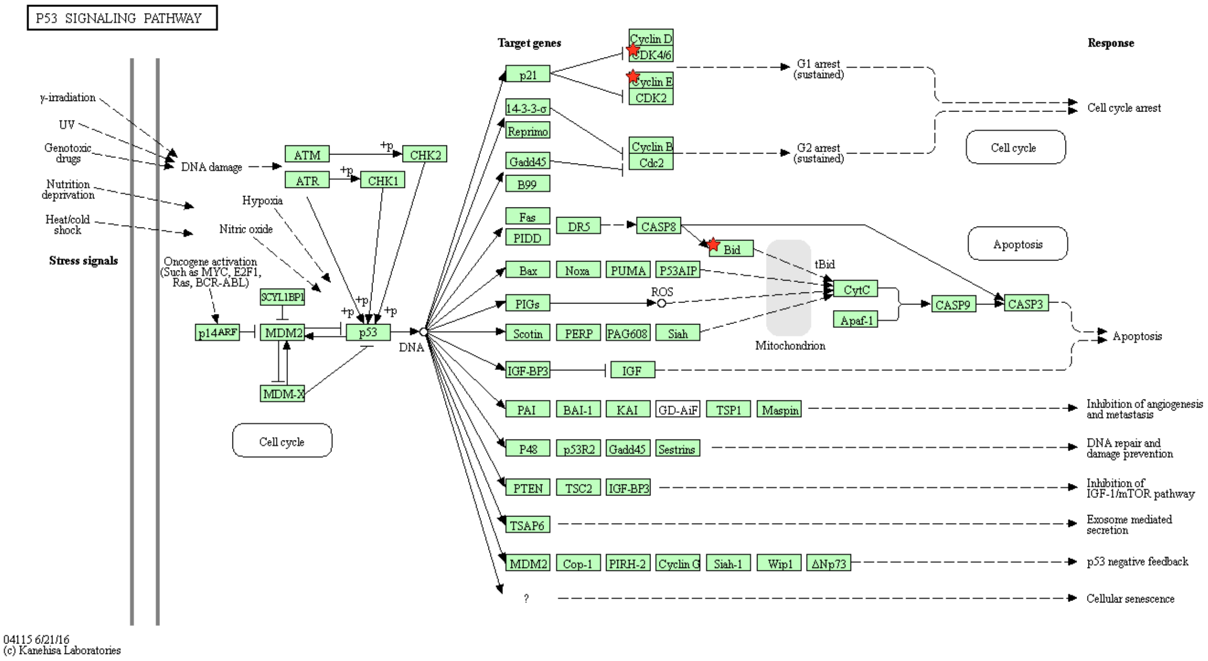

Supplementary Figure S6: Reprogramming of NBS fibroblasts into iPSCs shifts energy supply to *Glycolysis*.

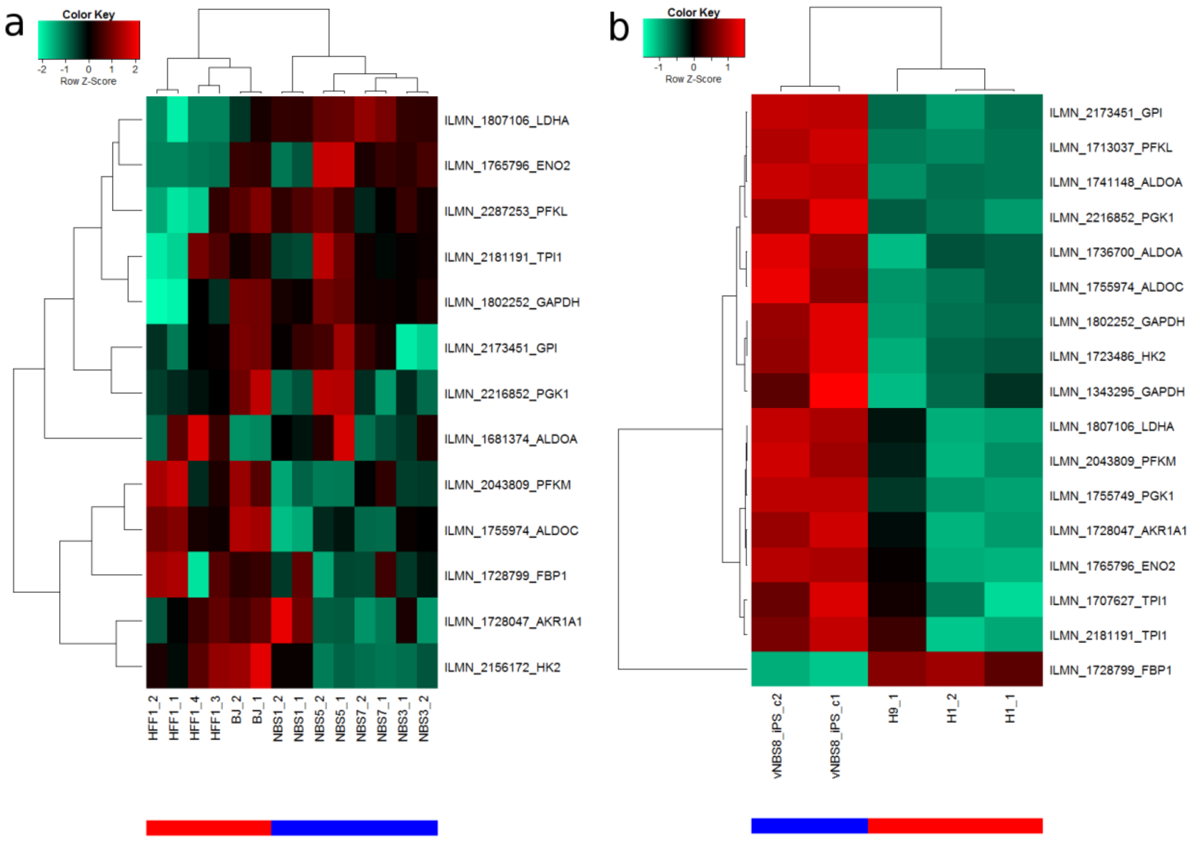

Supplementary Figure S7: Cluster analysis of *Oxidative phosphorylation* pathway in NBS fibroblasts and iPSCs.

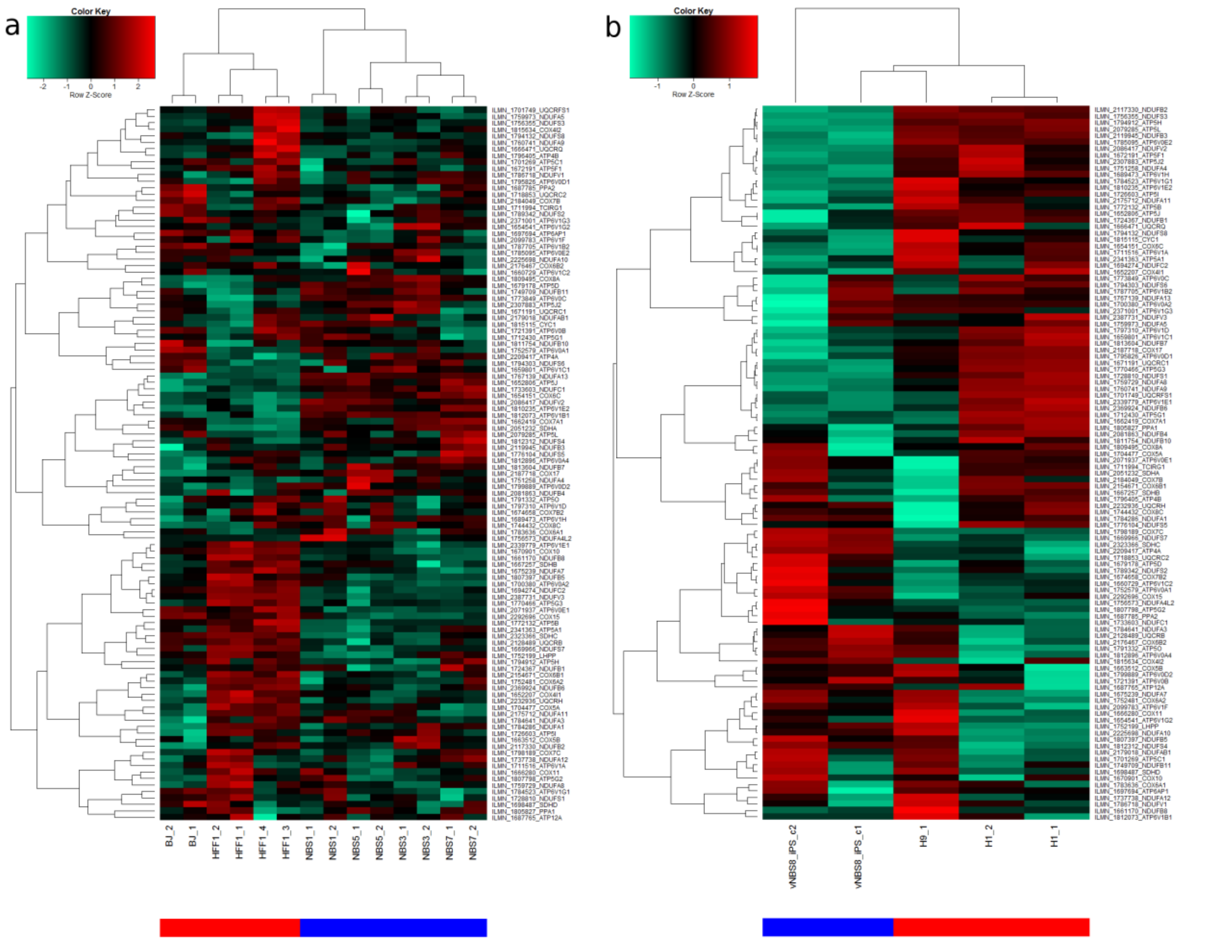

Supplementary Figure S8: Cluster analysis of *p53 signaling* pathway in NBS fibroblasts and iPSCs.

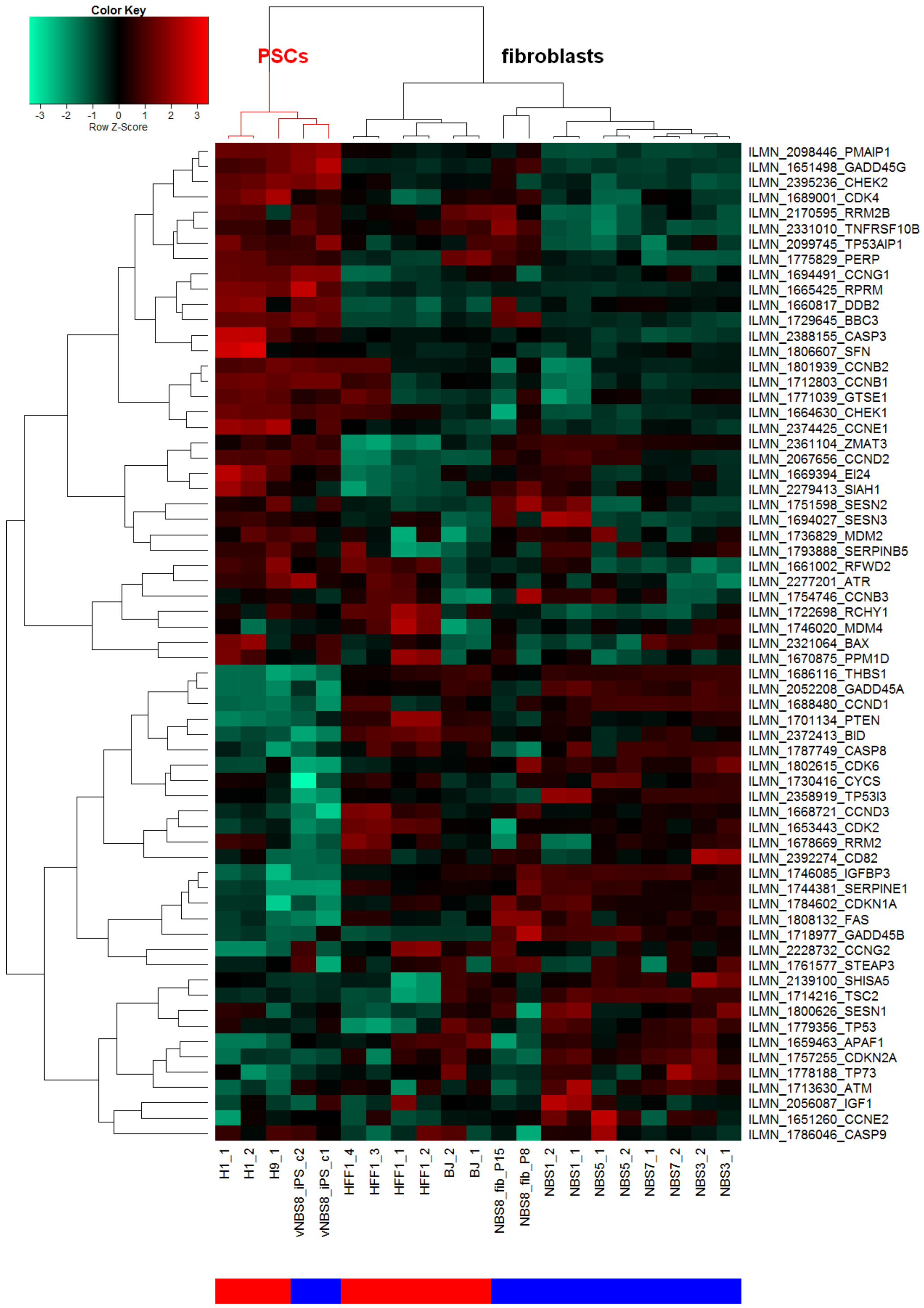

Supplementary table S1 to Supplementary Figure S3: Gene information

**PROBE_ID**	**SYMBOL**	**Ratio**	**EC Name**	**Definition**
ILMN_1728047	AKR1A1	1.57	1.1.1.2	aldo-keto reductase family 1, member A1
ILMN_1741148	ALDOA	2.49	4.1.2.13	aldolase A, fructose-bisphosphate
ILMN_1736700	ALDOA	2.03	4.1.2.13	aldolase A, fructose-bisphosphate
ILMN_1755974	ALDOC	2.70	4.1.2.13	aldolase C, fructose-bisphosphate
ILMN_1765796	ENO2	4.19	4.2.1.11	enolase 2 (gamma, neuronal)
ILMN_1343295	GAPDH	1.51	1.2.1.12	glyceraldehyde-3-phosphate dehydrogenase
ILMN_1802252	GAPDH	1.59	1.2.1.12	glyceraldehyde-3-phosphate dehydrogenase
ILMN_2173451	GPI	2.15	5.3.1.9	glucose-6-phosphate isomerase
ILMN_1723486	HK2	3.84	2.7.1.1	hexokinase 2
ILMN_1807106	LDHA	2.49	1.1.1.27	lactate dehydrogenase A
ILMN_1713037	PFKL	1.69	2.7.1.11	phosphofructokinase, liver
ILMN_2043809	PFKM	2.00	2.7.1.11	phosphofructokinase, muscle
ILMN_1755749	PGK1	2.45	2.7.2.3	phosphoglycerate kinase 1
ILMN_2216852	PGK1	1.78	2.7.2.3	phosphoglycerate kinase 1
ILMN_1707627	TPI1	1.62	5.3.1.1	triosephosphate isomerase 1
ILMN_2181191	TPI1	1.53	5.3.1.1	triosephosphate isomerase 1
ILMN_1728799	FBP1	0.39	3.1.3.11	fructose-1,6-bisphosphatase 1

Supplementary table S2: Refinement of genes differentially expressed in NBS-iPSCs vs. HESCs and annotated with pathways in cancer (DAVID funtional annotations)

Term	PValue	Genes	Benjamini
hsa05222:Small cell lung cancer	1.58E-12	CCNE1, E2F2, SKP2, NFKB1, CDK6, PIAS2, CDK4, LAMB1, MYC, ITGB1, PIK3R1, CHUK	1.02E-10
hsa05220:Chronic myeloid leukemia	8.93E-12	E2F2, CBLC, HRAS, CBLB, CTBP2, NFKB1, CDK6, CDK4, MYC, PIK3R1, CHUK	3.87E-10
hsa04151:PI3K-Akt signaling pathway	5.38E-11	FGF19, HRAS, FGF8, FGF11, KITLG, NFKB1, CDK6, FGF13, KIT, CDK4, ITGB1, CCNE1, VEGFA, LAMB1, MYC, PIK3R1, CHUK	1.75E-09
hsa05166:HTLV-I infection	1.79E-09	E2F2, HRAS, WNT10B, WNT3, SLC2A1, NFKB1, NFKB2, FZD5, CDK4, FZD4, MYC, CHUK, FZD7, PIK3R1	4.65E-08
hsa05212:Pancreatic cancer	3.61E-09	E2F2, RALBP1, VEGFA, TGFA, NFKB1, CDK6, CDK4, PIK3R1, CHUK	7.82E-08
hsa04014:Ras signaling pathway	5.20E-09	FGF19, HRAS, FGF8, RALBP1, RASSF1, VEGFA, FGF11, KITLG, NFKB1, FGF13, KIT, CHUK, PIK3R1	9.66E-08
hsa05218:Melanoma	7.41E-09	FGF19, E2F2, HRAS, FGF8, FGF11, FGF13, CDK6, CDK4, PIK3R1	1.20E-07
hsa05205:Proteoglycans in cancer	1.77E-08	CBLC, HRAS, CBLB, WNT10B, WNT3, VEGFA, FZD5, FZD4, MYC, ITGB1, FZD7, PIK3R1	2.56E-07
hsa05211:Renal cell carcinoma	9.75E-08	HRAS, VHL, VEGFA, SLC2A1, TCEB2, TGFA, EGLN1, PIK3R1	1.27E-06
hsa04550:Signaling pathways regulating pluripotency of stem cells	1.12E-07	BMP4, BMP2, HRAS, WNT10B, WNT3, FZD5, FZD4, MYC, FZD7, PIK3R1	1.33E-06
hsa05161:Hepatitis B	1.52E-07	CCNE1, E2F2, HRAS, BIRC5, NFKB1, CDK6, CDK4, MYC, PIK3R1, CHUK	1.65E-06
hsa04390:Hippo signaling pathway	2.16E-07	BMP4, BMP2, WNT10B, WNT3, RASSF1, BIRC5, FZD5, FZD4, MYC, FZD7	2.16E-06
hsa05217:Basal cell carcinoma	8.09E-07	BMP4, BMP2, WNT10B, WNT3, FZD5, FZD4, FZD7	7.51E-06
hsa05223:Non-small cell lung cancer	9.02E-07	E2F2, HRAS, RASSF1, TGFA, CDK6, CDK4, PIK3R1	7.82E-06
hsa04916:Melanogenesis	1.92E-06	HRAS, WNT10B, WNT3, KITLG, KIT, FZD5, FZD4, FZD7	1.56E-05
hsa04015:Rap1 signaling pathway	3.47E-06	FGF19, HRAS, FGF8, VEGFA, FGF11, KITLG, FGF13, KIT, ITGB1, PIK3R1	2.66E-05
hsa05219:Bladder cancer	4.14E-06	E2F2, HRAS, RASSF1, VEGFA, CDK4, MYC	2.99E-05
hsa05215:Prostate cancer	1.30E-05	CCNE1, E2F2, HRAS, TGFA, NFKB1, PIK3R1, CHUK	8.93E-05
hsa05221:Acute myeloid leukemia	1.97E-05	HRAS, NFKB1, KIT, MYC, PIK3R1, CHUK	1.28E-04
hsa04066:HIF-1 signaling pathway	2.42E-05	VHL, VEGFA, SLC2A1, TCEB2, NFKB1, EGLN1, PIK3R1	1.50E-04
hsa04660:T cell receptor signaling pathway	3.22E-05	CBLC, HRAS, CBLB, NFKB1, CDK4, PIK3R1, CHUK	1.90E-04
hsa05214:Glioma	4.10E-05	E2F2, HRAS, TGFA, CDK6, CDK4, PIK3R1	2.32E-04
hsa04010:MAPK signaling pathway	1.22E-04	FGF19, HRAS, FGF8, FGF11, FGF13, NFKB1, NFKB2, MYC, CHUK	6.62E-04
hsa04310:Wnt signaling pathway	1.65E-04	WNT10B, WNT3, CTBP2, FZD5, FZD4, MYC, FZD7	8.59E-04
hsa04012:ErbB signaling pathway	1.66E-04	CBLC, HRAS, CBLB, TGFA, MYC, PIK3R1	8.30E-04
hsa05203:Viral carcinogenesis	2.04E-04	CCNE1, HRAS, SKP2, NFKB1, CDK6, NFKB2, CDK4, PIK3R1	9.80E-04
hsa05206:MicroRNAs in cancer	2.63E-04	CCNE1, E2F2, HRAS, WNT3, RASSF1, VEGFA, NFKB1, CDK6, MYC	0.001222
hsa05230:Central carbon metabolism in cancer	5.75E-04	HRAS, SLC2A1, KIT, MYC, PIK3R1	0.002577
hsa05216:Thyroid cancer	6.84E-04	HRAS, TPR, MYC, TPM3	0.002959
hsa04110:Cell cycle	8.58E-04	CCNE1, E2F2, SKP2, CDK6, CDK4, MYC	0.003593
hsa05162:Measles	0.00118	CCNE1, NFKB1, CDK6, CDK4, PIK3R1, CHUK	0.004771
hsa04120:Ubiquitin mediated proteolysis	0.00134	CBLC, CBLB, VHL, TCEB2, SKP2, PIAS2	0.005282
hsa04810:Regulation of actin cytoskeleton	0.00158	FGF19, HRAS, FGF8, FGF11, FGF13, ITGB1, PIK3R1	0.006027
hsa05213:Endometrial cancer	0.00375	HRAS, MLH1, MYC, PIK3R1	0.013867
hsa05145:Toxoplasmosis	0.00548	NFKB1, LAMB1, ITGB1, PIK3R1, CHUK	0.019663
hsa05169:Epstein-Barr virus infection	0.00555	SKP2, NFKB1, NFKB2, MYC, PIK3R1, CHUK	0.01938
hsa04210:Apoptosis	0.00616	BID, NFKB1, PIK3R1, CHUK	0.020908
hsa05210:Colorectal cancer	0.00616	MLH1, BIRC5, MYC, PIK3R1	0.020908
hsa04115:p53 signaling pathway	0.00764	BID, CCNE1, CDK6, CDK4	0.025231
hsa04662:B cell receptor signaling pathway	0.00828	HRAS, NFKB1, PIK3R1, CHUK	0.02667
hsa05100:Bacterial invasion of epithelial cells	0.01158	CBLC, CBLB, ITGB1, PIK3R1	0.036263
